# Dataset model of academic boredom among junior high school students in Indonesian Islamic boarding schools

**DOI:** 10.3389/fpsyg.2026.1812545

**Published:** 2026-05-14

**Authors:** Ghozali Rusyid Affandi, Nur Ainy Fardana, Cholichul Hadi

**Affiliations:** 1 Doctoral Program of Psychology, Faculty of Psychology, Universitas Airlangga, Surabaya, Indonesia; 2 Psychology Study Program, Faculty of Psychology and Education, Universitas Muhammadiyah Sidoarjo, Sidoarjo, Indonesia

**Keywords:** academic boredom, Control-Value Theory of Achievement Emotions, dataset, education quality, Islamic boarding school, junior high school students

## 1 Introduction

Academic boredom remains a significant educational issue worldwide and in Indonesia (Affandi et al., 2023; Bekker et al., 2023). Academic boredom is defined as a negative emotional state characterized by a lack of interest, low motivation, and an inability to maintain attention during learning activities (Pekrun, 2024; Pekrun et al., 2010). Research shows that academic boredom can negatively impact students' academic performance, motivation, and psychological well-being (Goetz et al., 2020, 2019; Maier et al., 2023). In the context of Islamic boarding school education in Indonesia, this phenomenon becomes more complex because students not only face regular academic demands but also intensive religious education (Affandi et al., 2025a; Amria and Aulia, 2023).

This complexity is particularly pronounced in modern integrated Islamic boarding school (Type D)—the institutional context of the present study. Under the Ministry of Religious Affairs classification (Indonesia, 2019; Menteri Agama Republik Indonesia, 2006), Type D Islamic boarding schools integrate the full national curriculum with comprehensive Islamic studies (*dirosah islamiyah*) within a mandatory boarding system, in contrast to traditional Islamic boarding school (Types A–C), which focus exclusively on classical Islamic texts without a formal national curriculum. Consequently, students attend classes covering both general and Islamic subjects (*fiqh, hadith, nahwu sharaf* ) from morning to afternoon with limited break time, creating a higher academic burden than in single-curriculum settings (Alid et al., 2022; Chang et al., 2023; Lundeto et al., 2021). While this integrated system offers advantages, including structured spiritual formation, peer academic communities, and consistent mentorship by teaching staff as well as residential supervisors known as *musyrif* or *musyrifah*, it also presents significant challenges. The dense schedule, limited personal autonomy, and full day regulated routines from pre-dawn to nighttime elevate the risk of academic boredom, especially for early adolescents who need space for social exploration and identity formation (Ahmad et al., 2016; Suryana et al., 2022; Widiyanti and Affandi, 2025). These interacting dynamics are precisely what the present study seeks to explain.

The theoretical framework applied to comprehend these dynamics is the Control-Value Theory of Achievement Emotions (CVTAE) (Pekrun, 2024, 2006), which highlights that boredom stems from particular evaluations of control and value appraisals. Based on the recent development of CVTAE by Maier et al. (2023), this dataset focuses on the determinants at the micro and meso levels of the learning environment. Specifically, this dataset includes Cognitive Activation as a micro-level environmental factor, which refers to instructional methods that challenge students and stimulate cognitive engagement (Ekatushabe et al., 2022; Fauth et al., 2014; Maier et al., 2023). Additionally, this dataset includes Classroom Emotional Climate as a meso-level factor, which is defined as the collective perception of warmth, support, and positive interactions within the classroom (Hong et al., 2021; Meirovich, 2012).

Critically, in the Islamic boarding school context, these environmental factors are operationalized through a distinctive set of social agents: teachers, *musyrif/musyrifah* (dormitory supervisors), and administrative staff. Unlike regular school teachers, these agents interact with students throughout the entire day in classrooms, dormitories, and communal spaces. They provide both academic support, such as instruction, scaffolding, and formative feedback, and continuous emotional support through relational warmth, mentorship, and communal belonging. These two forms of support constitute key social environment inputs in the CVTAE framework that shape students' appraisals of control, or self-efficacy, and value, known as subjective task value. These are the two internal mediators through which environmental factors ultimately influence academic boredom (Maier et al., 2023; Pekrun, 2006; Reyes et al., 2012).

According to the model developed in this study, these environmental factors influence academic boredom through two mediators: self-efficacy (Affandi et al., 2025b; Bandura, 1997), which represents subjective control, and subjective task evaluation (Eccles and Wigfield, 2020; Part et al., 2020), which represents value judgment. Furthermore, given the conflicting evidence regarding the influence of gender on boredom (Feuchter and Preckel, 2023; Wang et al., 2022), this model specifically places gender as a control variable. This is done to isolate the pure influence of environmental and cognitive variables on boredom, regardless of students' biological or social differences. This control for gender is crucial to neutralize potential biases that may arise from strict educational segregation in Islamic boarding schools, as well as to address inconsistencies in previous literature regarding susceptibility to boredom between males and females (Feuchter and Preckel, 2023).

Despite growing scholarly attention to academic boredom, three significant research gaps remain. First, although CVTAE has been validated in Western and general educational settings, its applicability to non-Western, religiously integrated institutions such as Indonesian Islamic boarding schools, has not been tested using a validated multi-construct dataset that simultaneously captures environmental predictors, internal appraisal mediators, and demographic controls. Second, the mediation pathways through which cognitive activation and classroom emotional climate operate as antecedents of academic boredom have not been systematically examined within total institution settings, where academic and residential life are structurally inseparable. Third, most prior studies have not statistically controlled for gender as a structural variable. This represents a critical methodological omission in gender-segregated educational contexts such as Type D Islamic boarding schools, where classroom composition is institutionally determined (Feuchter and Preckel, 2023; Wang et al., 2022).

Based on the context described above, this article presents a dataset developed to validate the structural model of academic boredom in Islamic boarding schools based on CVTAE. The main purpose of presenting this data is to provide a comprehensive perspective on the influence of environmental and cognitive determinants on the academic boredom of junior high school students in Islamic boarding schools in Indonesia. Furthermore, the findings from this data are expected to serve as an empirical basis for the design of future educational interventions, particularly in overcoming boredom and improving the quality of the learning climate in the Islamic boarding school education system.

## 2 Method

### 2.1 Research design

This research employed a cross-sectional study design with a quantitative method to evaluate academic boredom and associated factors among junior high school students in Islamic boarding schools located in East Java, Indonesia. Ethical approval was obtained from the Ethics Committee of the Faculty of Psychology, Universitas Airlangga, with number 58/B/UN3.FPSI/III/TA.00.03/2025. Participation in this study was anonymous and voluntary, and all participants provided written consent before participating in the form of informed consent.

### 2.2 Participants and sampling

The participants in this research were junior high school students from Islamic boarding schools in East Java, aged between 12 and 16 years, with a total of 37,232 students. The minimum sample size based on Isaac and Michael's table with a confidence level of 99% was 663 students. A non-random sampling method was employed, combining purposive sampling and quota sampling techniques. The integration of purposive and quota sampling techniques offers an effective strategy in quantitative research, enabling researchers to flexibly select theoretically relevant respondents while ensuring a proportional representation of key population characteristics (Ahmed, 2024; Memon et al., 2025).

The initial stage employed purposive sampling to establish the sample criteria. The inclusion criteria for the selected sample were: (1) students aged between 12 and 16 years; (2) currently enrolled in grades 7, 8, or 9; (3) residing in Islamic boarding schools in East Java, Indonesia, for a minimum of 6 months, and (4) no prior diagnosis of psychological disorders. The second stage employed quota sampling to select 633 participants who met the inclusion criteria. Through direct data collection conducted at Islamic boarding schools in East Java, Indonesia using the paper-and-pencil method, data was gathered from a total of 740 students. After data cleaning, 642 out of 740 datasets were selected for inclusion in the measurement and modeling analysis. Demographic characteristics of the sample and psychometric properties of all instruments are presented in Table 1.

**Table 1:** Demographic profile and psychometric properties (*n* = 642).

Variables	Category	N	Percentage	Average academic boredom
Demographic Profile				
Gender	Male	326	50.78%	73.4
	Female	316	49.22%	71.23
Grade Level	Grade 7	281	43.77%	73.88
	Grade 8	235	36.60%	71.89
	Grade 9	126	19.63%	69.53
Age (years)	12	11	1.71%	79.55
	13	199	31.00%	72.55
	14	263	40.97%	72.72
	15	148	23.05%	71.04
	16	21	3.27%	69.67
Subjects	Natural science	109	16.98%	75.94
	Mathematics	133	20.72%	73.65
	Language	234	36.45%	72.37
	Islamic studies	109	16.98%	69.5
	Social science	48	7.48%	66.98
	Physical education	9	1.40%	68.67
**Variables**	**Measuring instrument**	**Item discrimination index**	**Cronbach's** α	**McDonald's** ω
Psychometric Properties				
Academic boredom	Academic boredom subscale (AEQ)	0.402–0.605	0.905	0.905
Cognitive activation	Cognitive activation subscale PISA 2012	0.177–0.608	0.723	0.678
Classroom emotional climate	Classroom emotional climate scale (CECS)	0.369–0.577	0.827	0.829
Subjective task value	Subjective task value scale	0.376–0.577	0.786	0.789
Self-efficacy	Self-efficacy questionnaire for children (SEQ-C)	0.45–0.682	0.922	0.922

### 2.3 Measuring instruments

The study utilized five scales adapted according to the guidelines of the International Test Commission (2017) and guidelines for the process of cross-cultural adaptation of self-report measures (Beaton et al., 2000), following three key stages: pre-condition, test development, and confirmation (Affandi et al., 2025a). All instruments were translated and adapted into Bahasa Indonesia, the language of instruction for national curriculum subjects in the participating schools, following a forward-backward translation procedure with bilingual expert review, per ITC Guidelines (International Test Commission, 2017) and Beaton et al. (2000). Construct validity was confirmed through CFA and item discrimination index testing; reliability was assessed via Cronbach's α and McDonald's ω. The measurement instruments employed in this research are as follows:

1) Cognitive Activation Scale adapted from the PISA 2012 Student Questionnaire Form B sub-scale (OECD, 2013), which is unidimensional with nine items that measure cognitive challenges in learning (CFI = 1.000, TLI = 1.000, RMSEA = 0.000; α = 0.722, ω = 0.724);
2) Classroom Emotional Climate Scale (CECS), adapted from the scale developed by (Hong et al. 2021), consisting of 12 items with four dimensions: teachers' academic support (three items), promotion of interaction (three items), promotion of mutual respect (three items), and respect for viewpoints (three items) (CFI = 0.951, TLI = 0.933, RMSEA = 0.059; α = 0.840, ω = 0.865);
3) Self-Efficacy Questionnaire for Children (SEQ-C), adapted from (Affandi et al. 2022) and (Muris 2001), covering the dimensions of academic self-efficacy (seven items), social self-efficacy (seven items), and emotional self-efficacy (seven items) (CFI = 0.999, TLI = 0.999, RMSEA = 0.008; α = 0.894, ω = 0.856);
4) Subjective Task Value Scale, adapted from the Expectancy-Value Theory developed by (Wigfield and Eccles 1992), consisting of three aspects: intrinsic interest value (two items), Attainment value/importance (three items), Utility value (two items) (CFI = 0.980, TLI = 0.961, RMSEA = 0.052; α = 0.730, ω = 0.799);
5) Academic Boredom Scale, adapted from a subscale of the Achievement Emotions Questionnaire (AEQ) developed by (Pekrun et al. 2011) consisting of four aspects: Affective (5 items), Cognitive (5 items), Motivational (5 items), and Physiological (7 items) (CFI = 0.919, TLI = 0.901, RMSEA = 0.071, ω = 0.917, α = 0.906) https://doi.org/10.17605/OSF.IO/GUBCD.

### 2.4 Data collection period and procedures

Data collection took place offline in classrooms during school hours from April to July 2025. The study focused on junior high school students from two Islamic boarding schools located in East Java, Indonesia. The survey was conducted using a paper-and-pencil method, directly supervised by the research team with support from the school's guidance counselors. Prior to questionnaire administration, the research team read standardized test instructions aloud, explaining the purpose of each scale, the Likert response format, and key constructs in age-appropriate language, while also informing participants of the research objectives, data anonymity, and their right to withdraw at any time without repercussions. Written informed consent was obtained from all students; since all participants were minors (aged 12–16 years), consent was additionally obtained from their parents or legal guardians, in compliance with applicable national regulations for research involving children and the Research Ethics Committee requirements. The questionnaire required approximately 45–60 min to complete. The entire research process was approved by the Research Ethics Committee of the Faculty of Psychology, Airlangga University (Approval No. 58/B/UN3.FPSI/III/TA.00.03/2025). As a token of appreciation, participants were given a stationery package upon completing the survey.

## 3 Data description

The dataset comprises survey responses from 642 students attending Islamic boarding schools in East Java. The full raw dataset, including item-level responses and instrument attachments, is publicly available under the title “Academic Boredom Survey: junior high school students in Indonesian Islamic Boarding Schools (*N* = 642)” and can be accessed via the following link: https://doi.org/10.17605/OSF.IO/GUBCD.

The data offers a comprehensive analysis of student response patterns for each item on the instrument, highlighting variations in their emotional experiences and perceptions of the learning environment. Detailed descriptive data can be seen in Table 2. Notably, the Academic Boredom scale reveals a significant occurrence of boredom-related symptoms. For instance, regarding the affective indicator (item Aff1), the majority of students selected “Agree” (42.5%) and “Strongly Agree” (14.3%), indicating they often felt bored during lessons. In contrast, only a small percentage (1.7%) chose “Strongly Disagree,” denying such feelings. A similar trend was observed with the cognitive indicator, where most students admitted to daydreaming or having their minds wander during class.

**Table 2:** Descriptive statistics: frequency and percentage.

Item/ response	1	2	3	4	5	6	7
Subjective task value							
	Very boring						Very interesting
IV1	14 (2.18)	33 (5.14)	83 (12.93)	135 (21.03)	130 (20.25)	81 (12.62)	166 (25.86)
	Not at all						Very much
IV2	156 (24.30)	119 (18.54)	132 (20.56)	121 (18.85)	67 (10.44)	35 (5.45)	12 (1.87)
	Not very beneficial						Very beneficial
AV3	8 (1.25)	17 (2.65)	51 (7.94)	62 (9.66)	100 (15.58)	102 (15.89)	302 (47.04)
	Not important						Very important
AV4	29 (4.52)	68 (10.59)	111 (17.29)	123 (19.16)	112 (17.45)	68 (10.59)	131 (20.41)
AV5	14 (2.18)	29 (4.52)	41 (6.39)	63 (9.81)	95 (14.80)	91 (14.17)	309 (48.13)
	Not very useful						Very useful
UV6	26 (4.05)	23 (3.58)	44 (6.85)	46 (7.17)	97 (15.11)	88 (13.71)	318 (49.53)
UV7	32 (4.98)	43 (6.70)	61 (9.50)	76 (11.84)	122 (19.00)	83 (12.93)	225 (35.05)
**Item/ response**	**1, very unsuitable**	**2, not appropriate**	**3, less suitable**	**4, somewhat suitable**	**5, appropriate**	**6, very appropriate**	**–**
Classroom emotional climate							
Tas1	21 (3.27)	56 (8.72)	96 (14.95)	195 (30.37)	206 (32.09)	68 (10.59)	–
Tas2	5 (0.78)	11 (1.71)	35 (5.45)	111 (17.29)	260 (40.50)	220 (34.27)	–
Tas3	5 (0.78)	22 (3.43)	74 (11.53)	136 (21.18)	264 (41.12)	141 (21.96)	–
Pi4	14 (2.18)	43 (6.70)	97 (15.11)	197 (30.69)	181 (28.19)	110 (17.13)	–
Pi5	14 (2.18)	31 (4.83)	86 (13.40)	166 (25.86)	222 (34.58)	123 (19.16)	–
Pi6	14 (2.18)	45 (7.01)	75 (11.68)	140 (21.81)	233 (36.29)	135 (21.03)	–
Pmr7	45 (7.01)	44 (6.85)	65 (10.13)	100 (15.58)	181 (28.19)	207 (32.24)	–
Pmr8	27 (4.21)	33 (5.14)	56 (8.72)	91 (14.17)	202 (31.46)	233 (36.29)	–
Pmr9	15 (2.34)	28 (4.36)	49 (7.63)	106 (16.51)	234 (36.45)	210 (32.71)	–
Rv10	9 (1.40)	7 (1.09)	35 (5.45)	118 (18.38)	234 (36.45)	239 (37.23)	–
Rv11	17 (2.65)	24 (3.74)	60 (9.35)	157 (24.46)	250 (38.94)	134 (20.87)	–
Rv12	26 (4.05)	62 (9.66)	104 (16.20)	184 (28.66)	186 (28.97)	80 (12.46)	–
**Item/ response**	**1, strongly disagree**	**2, disagree**	**3, somewhat agree**	**4, agree**	**5, strongly agree**	**–**	**–**
Academic boredom							
Aff1	11 (1.71)	42 (6.54)	224 (34.89)	273 (42.52)	92 (14.33)	–	–
Aff2	34 (5.30)	85 (13.24)	219 (34.11)	241 (37.54)	63 (9.81)	–	–
Aff14	113 (17.60)	147 (22.90)	194 (30.22)	108 (16.82)	80 (12.46)	–	–
Aff15	38 (5.92)	134 (20.87)	255 (39.72)	175 (27.26)	40 (6.23)	–	–
Aff16	108 (16.82)	226 (35.20)	170 (26.48)	104 (16.20)	34 (5.30)	–	–
Cog3	6 (0.94)	57 (8.88)	184 (28.66)	275 (42.84)	120 (18.69)	–	–
Cog4	19 (2.96)	92 (14.33)	130 (20.25)	243 (37.85)	158 (24.61)	–	–
Cog17	33 (5.14)	74 (11.53)	165 (25.70)	225 (35.05)	145 (22.59)	–	–
Cog18	27 (4.21)	137 (21.34)	196 (30.53)	215 (33.49)	67 (10.44)	–	–
Cog19	29 (4.52)	81 (12.62)	211 (32.87)	241 (37.54)	80 (12.46)	–	–
Mot5	136 (21.18)	220 (34.27)	156 (24.30)	73 (11.37)	57 (8.88)	–	–
Mot6	57 (8.88)	127 (19.78)	148 (23.05)	219 (34.11)	91 (14.18)	–	–
Mot7	51 (7.94)	126 (19.63)	157 (24.46)	177 (27.57)	131 (20.41)	–	–
Mot12	138 (21.50)	207 (32.24)	170 (26.48)	77 (11.99)	50 (7.79)	–	–
Mot13	114 (17.76)	166 (25.86)	175 (27.26)	113 (17.60)	74 (11.53)	–	–
Phys8	26 (4.05)	80 (12.46)	160 (24.92)	228 (35.51)	148 (23.05)	–	–
Phys9	40 (6.23)	117 (18.22)	166 (25.86)	176 (27.41)	143 (22.27)	–	–
Phys10	29 (4.52)	88 (13.71)	117 (18.22)	216 (33.64)	192 (29.91)	–	–
Phys11	23 (3.58)	58 (9.03)	139 (21.65)	251 (39.10)	171 (26.64)	–	–
Phys20	36 (5.61)	78 (12.15)	163 (25.39)	252 (39.25)	113 (17.60)	–	–
Phys21	50 (7.79)	140 (21.81)	208 (32.40)	167 (26.01)	77 (11.99)	–	–
Phys22	41 (6.39)	75 (11.68)	139 (21.65)	176 (27.41)	211 (32.87)	–	–
**Item/ response**	**1, Not at all**	**2, Poor**	**3, Good enough**	**4, Good**	**5, Very good**	**–**	**–**
Self-efficacy scale							
E1	19 (2.96)	118 (18.38)	260 (40.50)	142 (22.12)	103 (16.04)	–	–
E2	38 (5.92)	110 (17.13)	220 (34.27)	163 (25.39)	111 (17.29)	–	–
E3	22 (3.43)	123 (19.16)	246 (38.32)	180 (28.04)	71 (11.06)	–	–
E4	16 (2.49)	108 (16.82)	276 (42.99)	179 (27.88)	63 (9.81)	–	–
E5	29 (4.52)	109 (16.98)	184 (28.66)	155 (24.14)	165 (25.70)	–	–
E6	22 (3.43)	108 (16.82)	237 (36.92)	181 (28.19)	94 (14.64)	–	–
E7	18 (2.80)	121 (18.85)	261 (40.65)	164 (25.55)	78 (12.15)	–	–
A1	21 (3.27)	137 (21.34)	251 (39.10)	179 (27.88)	54 (8.41)	–	–
A2	24 (3.74)	179 (27.88)	252 (39.25)	142 (22.12)	45 (7.01)	–	–
A3	30 (4.67)	146 (22.74)	264 (41.12)	133 (20.72)	69 (10.75)	–	–
A4	30 (4.67)	182 (28.35)	247 (38.47)	145 (22.59)	38 (5.92)	–	–
A5	12 (1.87)	127 (19.78)	260 (40.50)	170 (26.48)	73 (11.37)	–	–
A6	16 (2.49)	90 (14.02)	209 (32.56)	188 (29.28)	139 (21.65)	–	–
A7	11 (1.71)	90 (14.02)	247 (38.47)	185 (28.82)	109 (16.98)	–	–
S1	22 (3.43)	123 (19.16)	247 (38.47)	180 (28.04)	70 (10.90)	–	–
S2	16 (2.49)	108 (16.82)	277 (43.15)	179 (27.88)	62 (9.66)	–	–
S3	29 (4.52)	109 (16.98)	185 (28.82)	155 (24.14)	164 (25.55)	–	–
S4	30 (4.67)	181 (28.19)	248 (38.63)	146 (22.74)	37 (5.76)	–	–
S5	12 (1.87)	126 (19.63)	261 (40.65)	171 (26.64)	72 (11.22)	–	–
S6	15 (2.34)	90 (14.02)	210 (32.71)	189 (29.44)	138 (21.50)	–	–
Item/ response	**1, never/ rarely**	**2, sometimes**	**3, often**	**4, always**	**–**	**–**	**–**
Cognitive activation							
CA1	93 (14.49)	239 (37.23)	224 (34.89)	85 (13.24)	–	–	–
CA2	44 (6.85)	205 (31.93)	275 (42.84)	117 (18.22)	–	–	–
CA3	126 (19.63)	243 (37.85)	180 (28.04)	92 (14.33)	–	–	–
CA4	85 (13.24)	229 (35.67)	226 (35.20)	100 (15.58)	–	–	–
CA5	64 (9.97)	230 (35.83)	235 (36.60)	112 (17.45)	–	–	–
CA6	30 (4.67)	146 (22.74)	277 (43.15)	188 (29.28)	–	–	–
CA7	114 (17.76)	204 (31.78)	228 (35.51)	94 (14.64)	–	–	–
CA8	33 (5.14)	155 (24.14)	267 (41.59)	186 (28.97)	–	–	–
CA9	98 (15.27)	242 (37.70)	216 (33.64)	85 (13.24)	–	–	–

Conversely, students' responses to predictor variables indicate a more favorable trend. Regarding the Cognitive Activation variable, a significant portion of students reported that teachers “Often” (34.9%) or “Sometimes” (37.2%) provided cognitive stimulation, such as encouraging critical thinking (item CA1). Similarly, perceptions of the Classroom Emotional Climate were generally positive, with 32% of students agreeing that statements about a supportive and positive climate were “Appropriate” for their classroom environment (item Tas1). This is reflected in the high levels of self-confidence among students; on the Self-Efficacy scale, a significant number rated their academic skills as “Good” (22.1%) or “Very Good” (16.0%) for item E1. Conversely, assessments of Subjective Task Value showed a relatively even distribution with a favorable trend, with option seven being the most frequently chosen.

## 4 Discussion

This dataset offers valuable empirical insights into the dynamics of academic emotions, framed through Pekrun's (2006) Control-Value Theory of Achievement Emotions (CVTAE). The data generated reveals that academic boredom is a multifaceted emotion, emerging from the interplay between students' personal traits and their learning environment. The finding of high levels of boredom in this dataset, even though students were in a perceived supportive environment, supports the literature stating that boredom is often a “silent emotion” that is less detected than other negative emotions such as anxiety (Maier et al., 2023; Pekrun et al., 2010). This data allows researchers to further explore why teaching strategies (cognitive activation) that are perceived as quite good by students have not been fully able to mitigate boredom in the boarding school context.

Furthermore, this dataset enriches the literature on the role of Classroom Emotional Climate (Hong et al., 2021; Reyes et al., 2012) in collective cultural contexts such as Islamic boarding schools. The high scores on self-efficacy and classroom climate perception in this data indicate that students have adequate psychological resources (“control”) and social support (Affandi et al., 2025b; Cui et al., 2017). According to Camacho-Morles et al. (2021) and Ekatushabe et al. (2022), the existence of these resources should be a protective factor against negative emotions. Therefore, the gap between high environmental support (descriptive data of independent variables) and high boredom (descriptive data of dependent variables) in this dataset offers an interesting anomaly that warrants further analysis by other researchers, for example, to test whether there are specific fatigue factors resulting from dormitory routines that affect students' value of academic tasks (Kristina and Jelena, 2024; Pekrun et al., 2010). The availability of item-level data is crucial for the development of more precise educational interventions to increase student engagement.

### 4.1 Limitations

This study has several limitations that should be acknowledged. First, due to its cross-sectional design, the data cannot establish causal relationships between variables. Longitudinal studies are required to better understand the direction and temporal dynamics of these relationships. Second, although researchers collaborated with school administrators to maximize participation, the sample was limited to only two Islamic boarding schools in East Java. This may restrict the generalizability of the findings to all Islamic boarding schools across Indonesia. Future research should explore the unique socio-demographic characteristics of students from other regions to provide a more comprehensive understanding.

Third, given the classroom-based nature of the research, most participants shared similar educational backgrounds. While the sample size satisfied the requirements for statistical power analysis, the relatively small number of participants in certain subject categories restricted the potential for meaningful comparisons across all subjects. Future research with larger, more diverse samples is recommended to enhance the generalizability of the findings.

Additionally, all data were gathered using self-report questionnaires, which are prone to response biases, including social desirability bias. Future research should utilize observational or objective measurement methods to better assess the validity of self-reported academic boredom. Additionally, this dataset lacks several potentially significant variables, such as actual academic performance, sleep quality, and family-related factors, which could impact academic boredom.

### 4.2 Data interpretation and reuse guidelines

This dataset can be utilized by researchers, practitioners, and policymakers interested in understanding academic boredom in Islamic boarding school contexts. The data support various analytical approaches including correlation analysis, regression modeling, structural equation modeling, and comparative studies across different populations. Users should consider the cross-sectional nature of the data when interpreting causal relationships and account for the specific cultural and educational context of Indonesian Islamic boarding schools. Importantly, these findings pertain specifically to modern integrated Islamic boarding schools (Type D) and should not be generalized to traditional Islamic boarding schools (Types A–C), regular schools, or non-Indonesian contexts without additional validation, given the substantial differences in curricular structure, residential support, and pedagogical approaches across these settings.

Filters applied during data collection included: (1) age restriction to 12–16 years, (2) minimum 6-month residence in boarding school, (3) active enrollment status, and (4) absence of diagnosed psychological disorders. These criteria should be considered when generalizing findings to other populations.

The dataset reflects the structural conditions that are unique to Type D Islamic boarding schools and that directly shape academic boredom risk: a dual curriculum, mandatory boarding, and full-day routines from pre-dawn to nighttime—conditions absent in both regular schools (single national curriculum, no boarding) and *salafiyah* Islamic boarding schools (religious curriculum only). These intersecting demands are particularly relevant when the perceived relevance of academic tasks is not actively reinforced by teachers and residential supervisors. The item-level data in this dataset allow practitioners to identify which boredom facets (affective, cognitive, motivational, physiological) predominate across subject contexts, enabling contextually targeted interventions; researchers should note that boredom profiles in other institutional types may differ substantially from those documented here.

## 5 Directions for future research

This dataset offers extensive opportunities for in-depth analysis and future research advancements:

1. **Exploring relationships and predictions:** researchers can leverage this dataset to conduct correlation tests or regression analyses, providing statistical evidence on how self-efficacy, task value, and classroom environment influence academic boredom, aligned with the principles of the CVTAE framework.
2. **In-depth psychometric analysis**: the available item-level data allows for Confirmatory Factor Analysis (CFA) or Rasch Model analysis to validate the structure of the Indonesian version of the academic boredom measurement tool.
3. **Comparative study**: researchers can collect comparative data from public school students (non-Islamic Boarding School) and use this dataset as a baseline to analyze differences in academic boredom profiles based on demographic factors.
4. **Intervention development**: based on descriptive profiles showing which items have the highest boredom scores, researchers can then design specific and targeted interventions to reduce student boredom.

## Data Availability

The datasets presented in this study can be found in online repositories. The names of the repository/repositories and accession number(s) can be found below: Open Science Framework: https://doi.org/10.17605/OSF.IO/GUBCD.
